# NFATc1 regulates the transcription of DNA damage-induced apoptosis suppressor

**DOI:** 10.1016/j.dib.2015.11.011

**Published:** 2015-11-17

**Authors:** Joo-Young Im, Kang-Woo Lee, Kyoung-Jae Won, Bo-Kyung Kim, Hyun Seung Ban, Sung-Hoon Yoon, Young-Ju Lee, Young-Joo Kim, Kyung-Bin Song, Misun Won

**Affiliations:** a Genome Structure Research Center, KRIBB, Daejeon 305-806, Republic of Korea; b Department of Biological Sciences, Korea Advanced Institute of Science and Technology (KAIST), Daejeon, 305-701, Republic of Korea; c Functional Genomics, Korea University of Science and Technology, Daejeon 305-350, Republic of Korea; d Biomedical Translational Research Center, KRIBB, Daejeon 305-806, Republic of Korea; e Genomics Research Center, KRIBB, Daejeon 305-806, Republic of Korea; f Department of Food Science and Technology, Chungnam National University, Daejon 305-764, Republic of Korea

**Keywords:** DDIAS, NFATc1, ChIP, Lung cancer, Pancreatic cancer

## Abstract

DNA damage induced apoptosis suppressor (DDIAS), or human Noxin (hNoxin), is strongly expressed in lung cancers. DDIAS knockdown induced apoptosis in non-small cell lung carcinoma A549 cells in response to DNA damage, indicating DDIAS as a potential therapeutic target in lung cancer. To understand the transcriptional regulation of *DDIAS*, we determined the transcription start site, promoter region, and transcription factor. We found that *DDIAS* transcription begins at nucleotide 212 upstream of the *DDIAS* translation start site. We cloned the *DDIAS* promoter region and identified NFAT2 as a major transcription factor (Im et al., 2016 [1]). We demonstrated that NFATc1 regulates DDIAS expression in both pancreatic cancer Panc-1 cells and lung cancer cells.

**Specifications Table**

**Table:** 

Subject area	Biology
More specific subject area	Cell biology, Molecular biology
Type of data	Image, graph, figure
How data was acquired	ChIP, Western blot, Luciferase assay
Data format	Raw
Experimental factors	Cells were overexpressed with NFATc1, NFATc2, or transfected with siRNA against NFATc1, NFATc2
Experimental features	Samples were HEK293, non-small cell lung cancer cells, NCI-H1703, A549 cells, and pancreatic cancer cells Panc-1
Data source location	Daejeon, Korea
Data accessibility	Data is available with the article

## **Value of the data**

• These data provide information regarding the transcription site and promoter used to control *DDIAS* transcription.
• NFATc1 plays a crucial role in *DDIAS* transcription in lung cancer cells.
• NFATc1 also controls DDIAS expression in the pancreatic cancer cell line PANC-1, as demonstrated by growth inhibition in a knockdown model.

## 1 Data

**Fig. 1:**
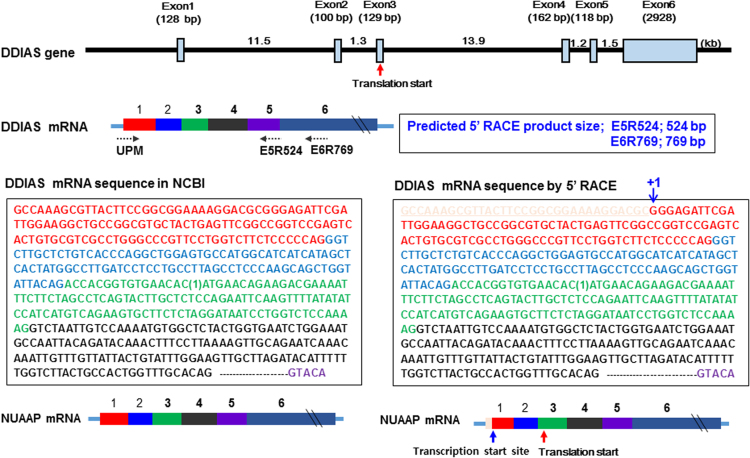
Determination of the transcription start site of DDIAS promoter. The transcription start site determined by 5**′**-RACE is 32 bp downstream of the site reported by the NCBI database.

**Fig. 2:**
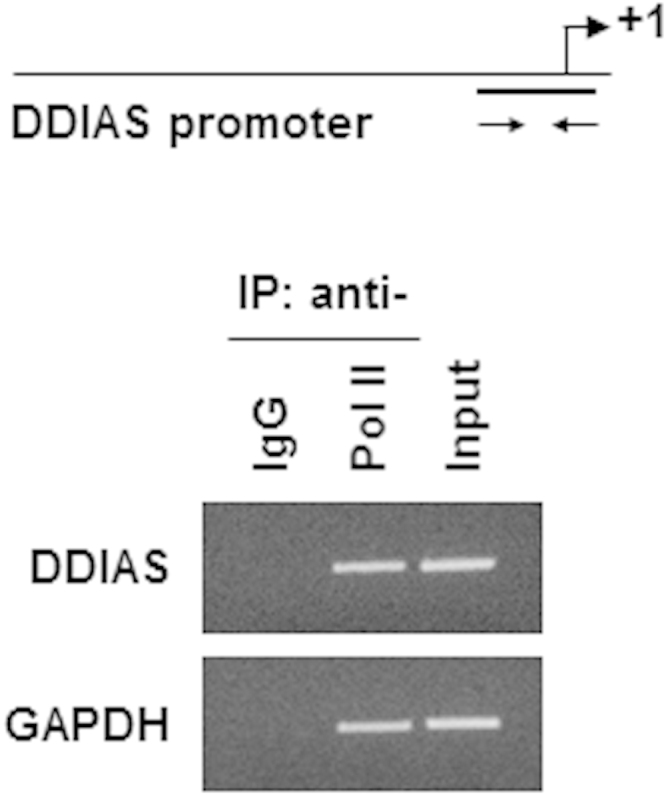
ChIP assay of RNA polymerase (ser5) in HEK293 cells. RNA polymerase II (phospho-ser5) binding to the *DDIAS* promoter in HEK293 cells. A ChIP assay was performed using an anti-RNA pol II antibody. An anti-IgG antibody was used as a negative control. PCR amplification was performed using the indicated *DDIAS* promoter-specific primers or control primers specific for *GAPDH*.

**Fig. 3:**
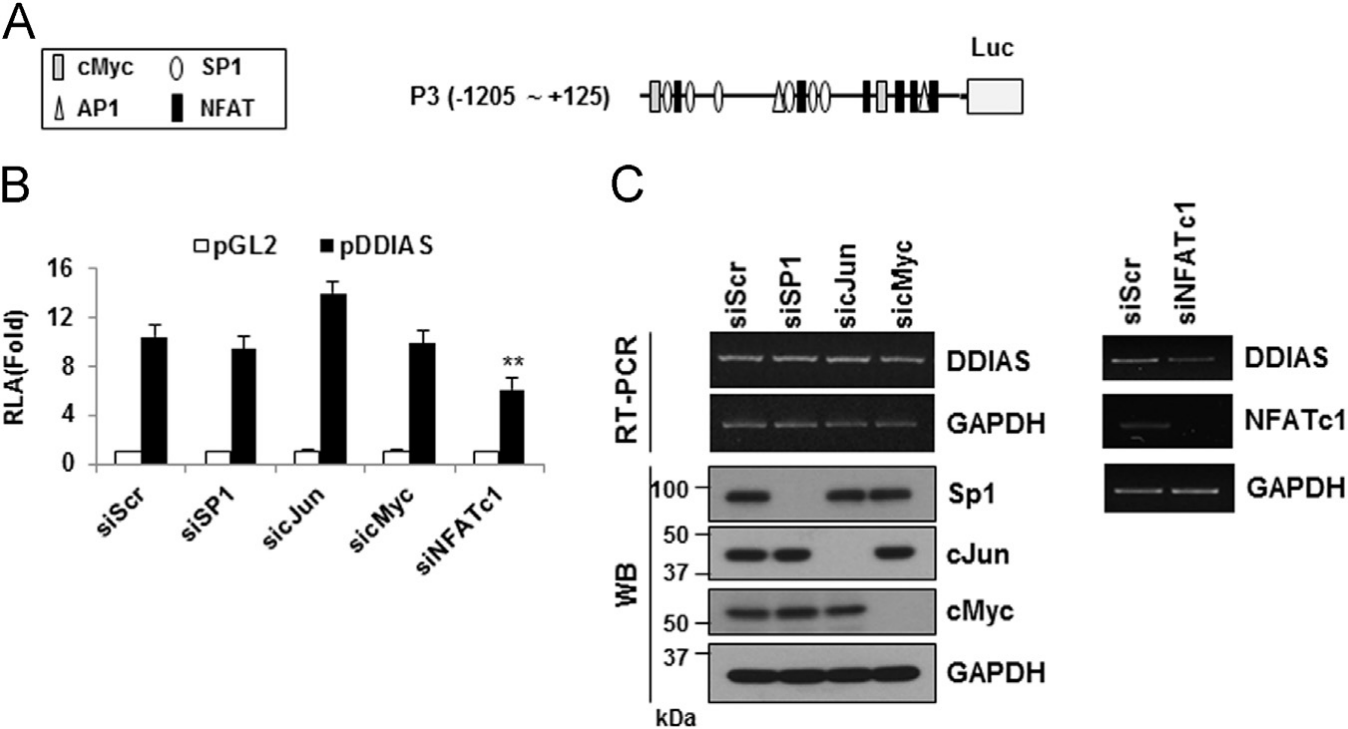
Putative transcription factor binding sites at P3 of the *DDIAS* promoter in HEK293 cells. (A) Putative transcription binding sites at P3 of the *DDIAS* promoter. (B–C) The effects of putative transcription factor knockdown were examined using a luciferase reporter assay (B); DDIAS mRNA and protein expression were evaluated using RT-PCR and Western blotting, respectively (C). HEK293 cells were transfected with siRNAs for SP1, cJun, and cMyc; scrambled siRNA was used as a control. All data are shown as means±standard errors of the mean (S.E.M).

**Fig. 4:**
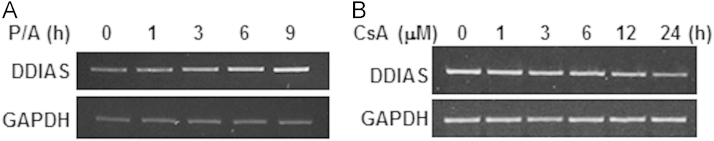
Regulation of *DDIAS* expression by NFAT in HEK293 cells. *DDIAS* mRNA level was examined after treatment of cells with PMA and A23187 (A) or CsA treatment (B).

**Fig. 5:**
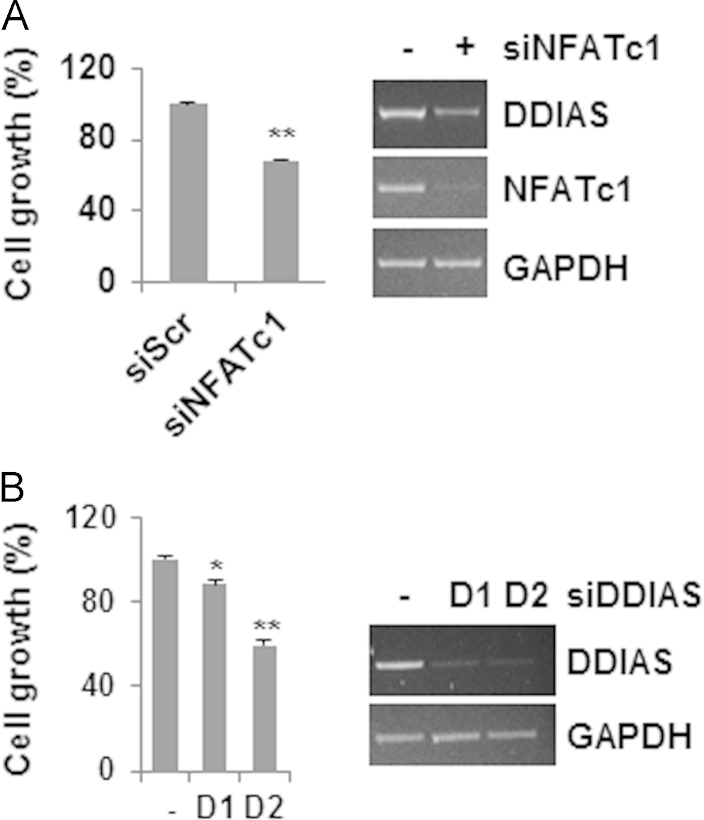
Effect of NFATc1 knockdown on DDIAS expression and cell growth in Panc-1 cells. Cells were transfected with siNFATc1 to induce NFATc1 knockdown. (A) Cell growth was determined after NFATc1 knockdown (siNFATc1 treatment for 96 h) using an SRB assay. All data are shown as means±S.E.M. **p<0.01. (B) The effect of DDIAS knockdown on cell growth determined by an SRB assay at 72 h. All data are shown as mean±S.E.M. *p<0.05 or **p<0.01.

A 5′-rapid amplification of cDNA ends (RACE) assay revealed a transcription start site located 212 nucleotides upstream of the *DDIAS* translation start site or 32 base pairs (bp) downstream of the site reported in the National Center for Biotechnology Information (NCBI) database (Fig. 1). This transcription start site was confirmed via RNA polymerase binding in a chromatin immunoprecipitation (ChIP) assay (Fig. 2). Putative transcription factor binding sites are located in the P3 region of the *DDIAS* promoter (Fig. 3A). In a P3-luciferase reporter assay, only NFATc1 knockdown significantly suppressed the promoter activity (Fig. 3B and C). *DDIAS* mRNA expression was found to be regulated by NFAT signaling, as shown by treatment with cyclosporine A (CsA), a calcineurin inhibitor, phorbol 12-myristate 13-acetate (PMA), and the calcium ionophore A23187 (Fig. 4). Treatment with PMA andA23187 increased the cellular expression of *DDIAS* mRNA (Fig. 4A). In contrast, the cellular *DDIAS* mRNA level decreased after treatment with CsA (Fig. 4B). Previously, we showed that DDIAS knockdown inhibits the growth of lung cancer cells [2]. In the pancreatic cancer cell line PANC-1, NFATc1 knockdown clearly induces DDIAS depletion and results in growth inhibition, indicating the NFATc1-mediated control of DDIAS expression (Fig. 5).

## 2 Experimental design, materials and methods

### 2.1 Determination of the transcription start site using 5′ rapid amplification of cDNA ends (RACE) in HEK293 cells

To identify the 5′ UTR of human *DDIAS*, 5′ RACE was performed using the SMART RACE cDNA Amplification Kit (Clontech, Mountain View, CA, USA) according to the manufacturer׳s protocol. First-strand cDNA was synthesized using RNA from human embryonic kidney 293 (HEK293) cells. 5′-RACE PCR was performed using the Advantage 2 PCR kit (Clontech) and the following gene-specific primers: R1; 5′-TGTACCTGTGCAAACCAGTGGCAGT-3′ or R2; 5′-AAAGCCTGCAATACCTGGGTCTGGT-3′. The PCR products were separated by electrophoresis on 1.5% agarose gels. After purification using the QIAquick Gel Extraction Kit (Qiagen, Valencia, CA, USA). PCR products were subcloned into the pGEM-T vector using the TA Cloning Kit (Promega, Madison, WI, USA) and sequenced. The first base pair after the adapter was identified as the transcription start site of the human *DDIAS* gene [1].

### 2.2 Cell culture and gene knockdown

HEK293, non-small cell lung cancer cell NCI-H1703, and pancreatic cancer cell Panc-1 cells were cultured in an incubator at 37 °C and 5% CO_2_. HEK293 and Panc-1 cells were cultured in Dulbecco׳s modified Eagle׳s medium (DMEM); NCI-H1703 cells were cultured in RPMI-1640 containing 10% fetal bovine serum (FBS), 50 U/mL of penicillin, and 50 μg/mL of streptomycin (Invitrogen, USA). Cells were transfected with 40 nM siRNAs with Lipofectamine 2000 (Invitrogen, USA) according to the manufacturer׳s instructions. siRNA sequences were as follows: siSP1, 5′-CUUUUUCACCAAUGCCAAU-3′, sicJun: 5′-CAAGAAGAUGCGCCGCAAC-3′, sicMyc: 5′-GUCAAGAGGCGAACACACA-3′, siNFATc1: 5′-GGACUCCAAGGUCAUUUUC-3′, siDDIAS D1: 5′-GCCUAUCAUUUCCCUGAUC-3′, siDDIAS D2: 5′-CUGUAACCCAGGCAGAUGU-3′, or siScrambled: 5′-CCUACGCCACCAAUUUCGU-3′.

### 2.3 Luciferase reporter assays

Cells were co-transfected with the *DDIAS* promoter (−1205) and pRL-TK, using 2 μl of Polyfect (Qiagen, USA) per well. Cells were harvested 36 h after transfection and lysed in 1× passive lysis buffer. Firefly and *Renilla* luciferase activities were assayed using a dual-luciferase reporter assay system (Promega, USA) and a luminometer (Victor X Light; Perkin Elmer, USA). Firefly luciferase activity was normalized to *Renilla* luciferase activity and expressed as the relative luciferase activity (RLA) to reflect promoter activity.

### 2.4 RT-PCR

Total RNA was extracted from cells using TRIzol (Invitrogen) and was reverse-transcribed into cDNA using TOPscript™ RT DryMIX (dT18) (Enzynomics, Daejeon, Korea) according to the manufacturer׳s instructions. The cycling conditions were 95 °C for 5 min, 95 °C for 30 s, 55 °C for 30 s, and 72 °C for 30 s. The following primers were used: NFATc1 F, 5′-GGGTTAAGTCCTCTCCCAAG-3′; R, 5′-GGGTTAAGTCCTCTCCCAAG-3′; DDIAS F, 5′-CTTGCAGCAGTTGTTACGAA-3′; R, 5′-GTGACCAAGCACTTCGAGTT-3′; GAPDH F, 5′-TCATGACCACAGTCCATGCC-3′; R, 5′-TCCACCACCCTGTTGCTGTA-3′.

### 2.5 Chromatin immunoprecipitation (ChIP) assay

ChIP assays were performed as previously described [3]. Cells were lysed and sonicated. The chromatin solution was incubated with 2 μg of anti-RNA polymerase II (phosphor-Ser5, #05-623B) or normal mouse immunoglobulin G (IgG; negative control, #12-371B) from ChIP assay kit (#17-371, Millipore, USA) overnight at 4 °C. These isolated DNA fragments were subsequently used as templates for a PCR analysis. Primers were used to amplify the putative NFAT consensus binding sites located in the DDIAS promoter region. The primers were as follows: forward for DDIAS, 5′-AAAAGGAGGCCAGCAGAAGCGC-3′; reverse for DDIAS, 5′- CCGCGTCCTTTTCCGCCGGAA-3′; forward for GAPDH, 5′-TACTAGCGGTTTTACGGGCG-3′; reverse for GAPDH, 5′-TCGAACAGGAGGAGCAGAGAGCGA -3′.

### 2.6 Immunoblotting assays

Cells were lysed with 1X RIPA buffer (Millipore) containing 1 mM Na_3_VO_4_, 1 mM sodium fluoride, 1 mM PMSF, and protease inhibitor cocktail (Roche, Switzerland). The lysates were quantified using a BCA assay kit (Bio-Rad, USA) and subjected to immunoblotting with specific antibodies, as follows: anti-SP1 (#5931) or anti-cJun (#9165) from Cell Signaling Technology (Beverly, USA), anti-cMyc (sc-40) or anti-NFATc1 (sc-7294 X) from Santa Cruz Biotechnology (Santa Cruz, USA), anti-GAPDH (LF-PA0212) from AbFrontier (Seoul, Korea). Antibodies were diluted in 5% BSA in phosphate-buffered saline containing 0.1% Tween 20 (PBS-T) overnight at 4 °C. After washing three times in PBS-T for 10 min, membranes were incubated for 1 h with horseradish peroxidase-labeled goat anti-mouse IgG (LF-SA8001) or goat anti-rabbit IgG (LF-SA8002) from AbFrontier and washed three times in PBS-T for 10 min. Western blot signals were detected using an enhanced chemiluminescence (ECL) kit (Millipore).

### 2.7 Cell viability

Cell viability was determined using the sulforhodamine B assay, as previously described [4]. The amount of sulforhodamine B dye bound to the cell matrix was quantified using a spectrophotometer at 530 nm.
